# A Multireference Picture of Electronic Excited States in Vanadyl and Copper Tetraphenyl Porphyrin Molecular Qubits

**DOI:** 10.1021/acs.jpca.5c03946

**Published:** 2025-07-29

**Authors:** Arup Sarkar, Alessandro Lunghi

**Affiliations:** School of Physics, AMBER and CRANN Institute, 8809 Trinity College Dublin D2, Ireland

## Abstract

The nature of electronic excited states has a deep impact on the dynamics of molecular spins but remains poorly understood and characterized. Here we carry out a thorough multiconfigurational investigation for two prototypical molecular qubits based on vanadyl and copper tetraphenyl porphyrins. State-average CASSCF and NEVPT2 calculations have been employed with four different active spaces of growing complexity to account for the d–d, second d-shell, ligand-to-metal charge transfer states, and π–π* excited states, revealing an in-depth picture of low-lying excited states in agreement with experimental observations. The largest active spaces attempted (13,14) for the vanadyl and (17,12) for the copper compounds reveal that the lowest-lying excited states originate from π–π* quartet excitations. These findings shed light on the nature of the excited states of molecular qubits, taking an important step toward elucidating their role in molecular spin dynamics as well as determining novel strategies for the optical read-out of spin states.

## Graphical abstract

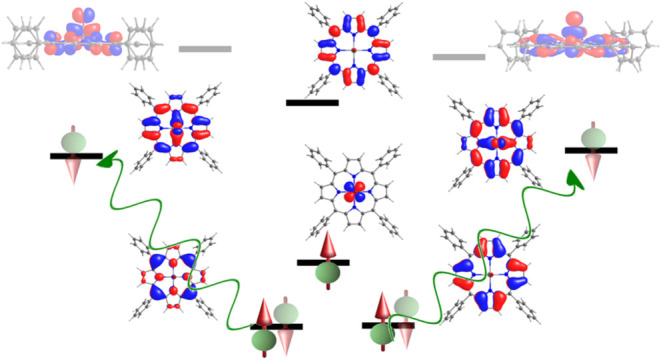

## Introduction

Porphyrins are planar macrocyclic aromatic compounds with largely delocalized π-electron clouds widely used in optoelectronics due to their tunable optical and redox properties. Their rigid structure and ability to coordinate with both diamagnetic and paramagnetic metal ions make them ideal for a range of photophysical applications, including nonlinear optics,^1^ light-harvesting,^2^ chemical/biological sensing,^3^ photodynamic therapy,^4,5^ and photocatalytic applications.^6^ Moreover, porphyrins, and in particular tetraphenylporphyrins (TPPs), exhibit long spin coherence lifetimes when coordinated with spin-1/2 metal ions such as vanadyl (VO^2+^) or cupric (Cu^2+^) ones (see Figure 1), making them appealing for quantum information processing applications^7–12^ and building hybrid spin-optical quantum interfaces.^13,14^

**Figure 1:**
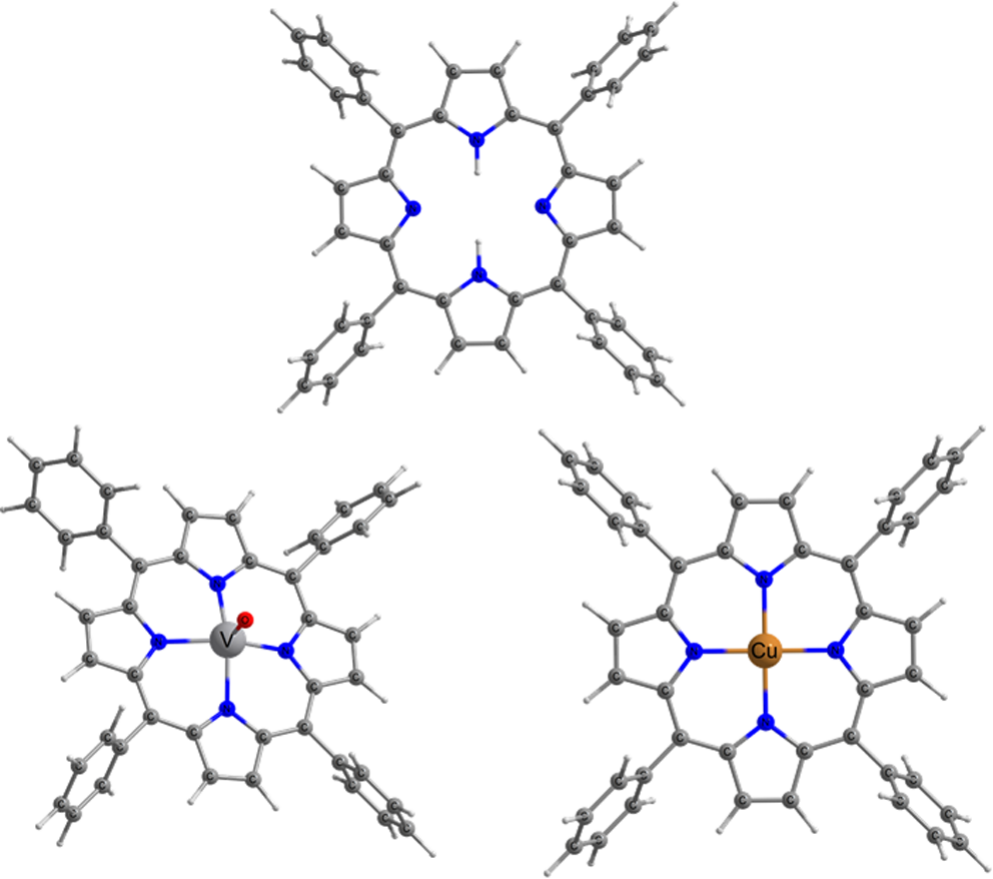
Molecular representation of H_2_TPP (top), VOTPP (bottom left), and CuTPP (bottom right).

In the context of quantum information science, maximizing the coherence, *T*_2_ and lifetime, *T*_1_ of molecular spin qubits has so far been a priority and a plethora of spin-1/2 systems containing VO^2+^ or V^4+^ and Cu^2+^ complexes have been investigated over the past decade to interpret the impact of solvents,^15^ nuclear spin,^16^ metal–ligand covalency,^17,18^ ligand-field symmetry,^19,20^ and d-d excitations^21^ on these time constants. Despite the great deal of knowledge produced by these studies, the role of electronic excitations, other than d–d excitations, on spin dynamics remains poorly understood. This represents an outstanding knowledge gap with potentially large repercussions on our ability to engineer molecular qubits with improved functionalities. Indeed, both theoretical and experimental evidence for the importance of excited states in determining the relaxation rate of molecule spins has built over the past few years,^20–23^ and a recent ab initio study has demonstrated that electronic excited states are key in promoting Raman relaxation starting from already ∼20 K and up to room temperature.^24^ The study of electronic excitations in molecular qubits is made even more urgent considering emerging studies on spin-optical interfaces as a means to initialize and read out molecular spins.^13^ In both these examples, the energy position and nature of the low-lying electronic excitations are of paramount importance in dictating the values of *T*_1_,^24^ as well as the probability to observe optical emission instead of vibrational relaxation.^25^

Over the past few decades, the development of high-performance computing has made multiconfiguration (MCSCF) quantum chemical methods a routine choice for describing excited states and correlated wave functions of open-shell systems.^26,27^ These methods, combined with second-order perturbative corrections (PT2) or other post-MCSCF methods, not only provide an unambiguous description of metal-to-metal electronic excitations, but are also able to accurately estimate magnetic properties such as zero-field splitting, ligand-field interactions, and spin-Hamiltonian parameters in transition metals, lanthanide, and actinide complexes.^28^ Addressing the true nature of molecular excited states, however, requires going beyond simulating those metal-to-metal transitions. Attempts in incorporating metal–ligand covalency effects have also been done by including the effect of second d-shell,^29–33^ and ligand-based aromatic/delocalized π-orbitals^31,34–36^ in small to medium-sized transition metal complexes using various multireference wave function-based approaches. The complexity of such a task is well exemplified by Serrano-Andres’ et al.,^37^ Pierloot et al.,^38,39^ Manni et al.,^40,41^ and Zhou et al.,^42^ who tested a variety of multiconfiguration methods, such as complete active space self-consistent field (CASSCF), complete active space second-order perturbation theory (CASPT2), restricted active space self-consistent field (RASSCF), Full Configuration Interaction Quantum Monte Carlo (FCIQMC), and Multiconfiguration Pair-Density Functional Theory (MC-PDFT) with different active spaces, to explore the excited states of porphyrin and Fe-porphyrin systems. Importantly, while some insights into the optical excitations in Cu­(II) porphyrins have recently been reported,^31^ no comprehensive study of electronic excitations is available for VO-based compounds. Given the central position of such class of molecules in the field of magnetic molecules for quantum information science,^8^ filling such a knowledge gap is particularly urgent.

Unlike the porphyrins, which are quite symmetric, here, we build on this literature to address the following key aspects of the VOTPP and CuTPP’s (preserving the four phenyl groups) molecular excited states: (i) identify the nature of low-lying excited states, (ii) determine how the ligand-to-metal charge transfer (LMCT) states, metal-based d-d excitations, and ligand-based π–π* excitations combined describe the absorption spectra, and (iii) determine the effect of these excitations on the spin–orbit coupling and electronic *g*-factors. Our work provides an in-depth quantitative depiction of excited electronic states of TPP-based molecular qubits and establishes a solid foundation for spin relaxation theory and the engineering of molecular spin-optical interfaces.

## Computational Methods

All the calculations are performed using the ORCA 5.0.4 quantum chemistry package.^43^ Starting geometries of VOTPP and CuTPP are taken from the X-ray structures reported by Yamabayashi et al.^10^ and Urtizberea et al.,^7^ respectively. Gas-phase geometry optimizations for H_2_TPP, VOTPP, and CuTPP molecules are carried out using BP86 ^44^ functional with Grimme’s D3 dispersion correction^45^ and Becke-Johnson damping.^46^ Def2 basis sets of the Karlsruhe group with split valence polarization function^47^ are used for all the elements. The second-order scalar relativistic Douglas-Kroll-Hess^48^ Hamiltonian is employed during all the multireference calculations. DKH-contracted basis sets have been used for different atoms- DKH-def2-TZVP^47^ basis set for the V and Cu centers, DKH-def2-TZVP­(-f) for O, N, and C atoms, and DKH-def2-SVP basis set for H. The initial guess orbitals for the CASSCF calculations are generated using restricted open-shell Kohn–Sham (ROKS) or unrestricted KS (utilizing quasi-restricted orbitals) calculations. During the state-average CASSCF calculations, different active spaces are chosen for the H_2_TPP, VOTPP, and CuTPP molecules based on the 3d, 4d, and ligand-based σ and π orbitals (see Table 1). The dimension of the guess matrix was increased to 4000 to improve the convergence in the CI step for handling large active spaces. The *N*-electron valence state perturbation theory second order (NEVPT2)^49^ is employed to account for the dynamic electron correlation. Spin–orbit interactions, and subsequent *g*-factors are computed using the quai-degenerate perturbation theory (QDPT) combined with the effective Hamiltonian approach (EHA) using the spin–orbit mean field (SOMF) integral.^50^

**Table 1:** Active Space Composition of the Three Molecules

	AS1	AS2	AS3	AS4
H_2_TPP	(4,5)	(8,7)	-	-
	3d	3d + 4d	3d + first coordination sphere σ/π	3d + delocalized π
VOTPP	(1,5)	(1,10)	(9,9)	(13,14)
CuTPP	(9,5)	(9,10)	(11,6)	(17,12)

### Active Space Selection

For the H_2_TPP molecule, the smallest active space (AS1) includes four electrons in five π orbitals, i.e., (4,5), comprising highest occupied molecular orbital-1 (HOMO–1), HOMO, lowest unoccupied molecular orbital (LUMO), LUMO + 1, and LUMO + 2 orbitals. These five π orbitals cover the carbons and nitrogens of the central porphyrin ring, excluding the four substituted phenyl groups. In the next step (AS2), two more π orbitals are added from the occupied orbitals, making it the (8,7) active space (see Table 1). The latter two π orbitals belong to the b_2g_ and b_3g_ point group symmetries from the *D*_2*h*_ point group which tends to interact with the *d*_*xz*_ and *d*_*yz*_ set of the metal 3d orbitals, as discussed later in more detail.

The active spaces for the VOTPP and CuTPP molecules are similarly built by using a systematic increase in their complexity. The minimal active space consists of the five metal 3d orbitals (AS1) and 3d electrons, namely, (1,5) and (9,5) for Vanadyl (VO^2+^) and Cu^2+^ ions, respectively. In addition to 3d, the inclusion of the second d-shell, i.e., the 4d (or 3d′), is also tested for these two complexes, leading to the model AS2. Next, in AS3 and AS4, ligand σ and π orbitals are added to the 3d space to incorporate the ligand-to-metal, metal-to-ligand, and ligand-to-ligand excitations. In AS3, a (9,9) active space is chosen for the (VO^2+^) species to accommodate the vanadium 3d and oxygen 2p-based bonding orbitals which constitute d_*z* ^2^_-p*_z_* (σ) and d*_xz_*-p*_x_*/d*_yz_*-p_*y*_ (π) molecular orbitals (MOs). In the case of CuTPP, this AS3 is restricted to (11,6) by incorporating the bonding counterpart of the d_*x* ^2^–*y* ^2^_ orbital consisting of the N-2p_*x*_/p_*y*_ atomic orbitals. The AS4 constitutes the largest active space for these two complexes, which accounts for the delocalized π-orbitals from the porphyrin ring. In the case of VOTPP, the AS4 or (13,14) consists of all the (9,9) active space orbitals along with the (4,5) active space from TPP MOs. Now, the other two doubly occupied π orbitals, which were present in the (8,7) active space of H_2_TPP, are not close-lying to make bonding interactions with the vanadium d_*xz*_ or d_*yz*_ orbitals. For CuTPP, AS4 consists of (9,5) with the (4,5) active space from H_2_TPP and two more doubly occupied π orbitals from the porphyrin ring, making it (17,12). These two active spaces (AS4) are certainly not the largest possible active space for this system; however, from the standpoint of state-average calculations with low-lying excited states, they can be considered an optimal choice within the CASSCF limit.

## Results

### Free Tetraphenyl Porphyrin Ring

At first, the electronic excited states for the neutral ligand framework are examined. The ground state of the free tetraphenyl porphyrin (H_2_TPP) is a closed-shell singlet with valence molecular orbitals (MOs) consisting of delocalized π electrons spread over the porphyrin ring’s C and N atoms. These conjugated π electronic systems are constructed by the linear combination of C-2p_*z*_ and N-2p_*z*_ atomic orbitals (see Figure 2). The absorption spectrum of the free base H_2_TPP mainly originates from singlet to singlet π–π* transitions within the porphyrin ring (see Figure 2). Experimentally, two absorption bands are observed in the visible region, namely, Q_*x*_ (1.91–2.07 eV or 600–650 nm) and Q_*y*_ (2.25- 2.42 eV or 512–550 nm) bands.^40,51–53^ Experimentally, four peaks are observed for these Q_*x*_ and Q_*y*_ bands due to symmetry breaking or vibrational coupling. The other band, generally referred to as the Soret band, always appears near the ultraviolet (UV) region around 3.10–3.33 eV (400–372 nm), and presents the strongest absorption intensity.

**Figure 2:**
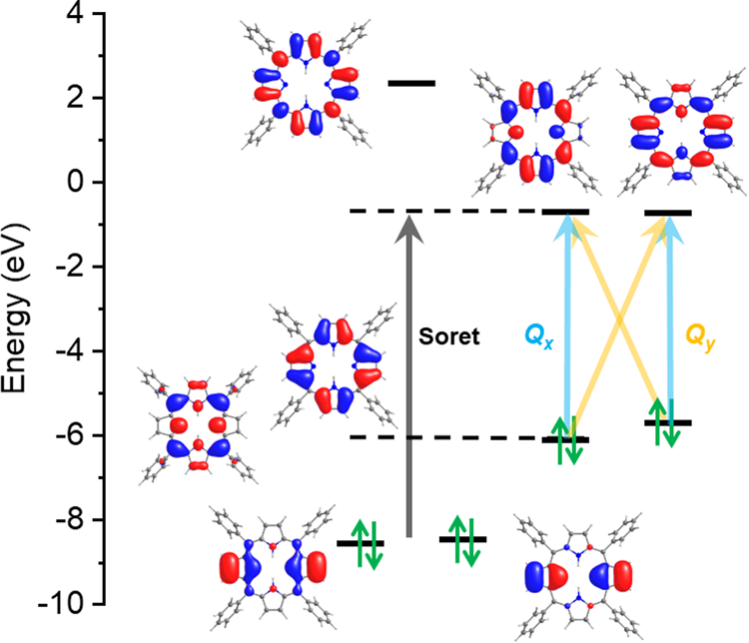
Absolute energies of the state-average orbitals of H_2_TPP obtained from CASSCF­(8,7) calculations. The electronic occupation corresponds to the ground-state electron configuration. The four Gouterman’s orbitals are HOMO, HOMO–1 and LUMO, LUMO + 1.

The NEVPT2 computed excitation energies using the (8,7) active space with 10 triplets and 10 singlet states (below 4 eV energy cutoff) efficiently capture all three excitation bands and display a nice agreement with the experimental values (see Table 2). Multiconfiguration wave function analysis reveals that the Q_*x*_ and Q_*y*_ bands originate from the electronic transition from the two doubly occupied π orbitals to the two antibonding π* pair of orbitals (see Figure 2). On the other hand, the high-energy Soret band arises from the dominant electronic transition from the lower-lying π-bonding orbitals (HOMO–2 or HOMO–3) to the first excited π* pairs. Interestingly, relative to the ground state singlet, the first excited state is found to be a triplet, located at 1.57 eV, compared to the first excited singlet, which is at 1.85 eV as obtained from the NEVPT2 calculations (Table 2). The CASSCF electronic configurations (or occupations) shown in Table 2 follow the same order as that depicted in Figure 2. Compared to NEVPT2 excitation energies, SA-CASSCF overestimates the excited states by more than 1 eV (Tables S1 and S2 in the SI). The (4,5) active space lacks the two lowest occupied MOs from the (8,7) one, thus missing the Soret band among its solutions. However, the Q_*x*_ and Q_*y*_ bands are predicted in a similar region as compared to the (8,7) active space from NEVPT2 calculations (see Table S3). Notably, the Q_*x*_ and Q_*y*_ bands approach closer to each other when the acidic hydrogen atoms are removed to make it a dianion (see Table S4 in the SI), in virtue of an overall higher symmetry of the complex.

**Table 2:** Vertical Excitation Energies and Wave Function Decomposition of H_2_TPP from SA-CASSCF/NEVPT2 Calculations^a^

singlet states	CASSCF wave function (major weightage)	NEVPT2 (CASSCF) energy (eV)	bands (NEVPT2 osc. Str.)	exp. (eV)
GS	2 2 2 2 0 0 0 (91%)	0.0		
first ES	2 2 u 2 0 d 0 (49%)	1.85 (2.88)	Q_*x*_ (0.02)	1.91–
	2 2 2 u d 0 0 (43%)			2.07
second ES	2 2 2 u 0 d 0 (49%)	2.29 (3.20)	Q_*y*_ (0.01)	2.25–
	2 2 u 2 d 0 0 (44%)			2.42
third ES	u 2 2 2 d 0 0 (29%)	2.98 (5.15)	Soret (1.46)	3.10–3.33
	2 2 u 2 d 0 0 (28%)			
	2 2 2 u 0 d 0 (25%)			
fourth ES	u 2 2 2 0 d 0 (49%)	3.00 (4.22)	Soret (0.2)	
	2 2 2 u d 0 0 (19%)			
fifth ES	2 u 2 2 0 d 0 (78%)	3.10 (4.19)	-	
sixth ES	u 2 2 2 d 0 0 (46%)	3.17 (4.51)	Soret (0.2)	
	2 2 u 2 d 0 0 (18%)			
Triplet States				
first ES	2 2 u 2 u 0 0 (58%)	1.57 (2.42)		
	2 2 2 u 0 u 0 (35%)			
second ES	2 2 u 2 0 u 0 (87%)	1.83 (2.85)		
third ES	2 2 2 u 0 u 0 (60%)	1.91 (2.61)		
	2 2 u 2 u 0 0 (34%)			
fourth ES	2 2 2 u u 0 0 (90%)	2.15 (2.46)		

a Only major determinants (u = spin up, d = spin down) are shown; conjugate determinants are omitteds.

### Vanadyl Tetraphenyl Porphyrin

Next, we investigated the excited states of the VOTPP molecule. At first, the minimal active space (AS1) consists of only the 3D-metal orbitals used to compute the five excited states within the doublet spin multiplicity. The metal-centered orbitals are well separated due to the strong ligand field of the V­(IV) ion, and the highest-energy d_*z* ^2^_ orbital lies 4.5 eV apart relative to the singly occupied d_*xy*_ orbital (see Table S5 and Figure S1 in the SI). Adding the second d-shell into the active space (AS2) barely changes the excitation energies of the VOTPP molecule (Table S6 in the SI). Expanding the active space onto the axial oxygen-based p_*x*_, p_*y*_, p_*z*_, and the nitrogen-based σ_*x* ^2^–*y* ^2^_ orbitals gives rise to (9,9) active space which are also the bonding counterpart of the vanadyl d_*xz*_, d_*yz*_, d_*z* ^2^_, and d_*x* ^2^–*y* ^2^_ antibonding orbitals, respectively (see Figure S2 in the SI). The inclusion of these four bonding orbitals allows one to identify ligand-to-metal charge transfer (LMCT) states along with the d–d transitions. The low-lying LMCT states involve the electronic transition from the oxygen-based π_*xz*_/π_*yz*_ orbitals to the vanadium-centered d_*xz*_/d_*yz*_ orbitals (see Table S7 in the SI). These LMCT excited states appear from 5.6 eV and are significantly higher in energy than the d–d excited states.

At this point, it is quite clear that the state-average calculations using (1,5) and (1,10) active spaces for VOTPP capture only the excited states based on the metal d-orbitals and do not address the low-lying excited states or Q-bands correspond to the π–π* excitations which are prominent in the H_2_TPP molecule below 2 eV energy window. The (9,9) active space and the associated excited states, on the other hand, provide insights regarding the high-energy LMCT states, primarily localized on the V–O π-orbitals. However, none of these active spaces involve the porphyrin π orbitals. Moreover, it is crucial to determine whether d-d excitations are the only spin-allowed and low-lying excited states that appear within 3 eV or if other ligand-centric (π–π* or metal-to-ligand charge transfer) transitions also interfere with the d-d transition energies. In order to capture the complete ligand and metal-based electronic states, a large active space is constructed to address these questions by adding the delocalized ligand (or TPP) π orbitals along with the existing V–O d/π-orbitals. Thus, the (13,14) active space is founded by combining the vanadyl (9,9) active space with the TPP (4,5) active space (Figure 3). Both state-specific (with only ground state root) and state-average calculations have been performed for the VOTPP molecule (see Table 3). State-specific CASSCF­(13,14) orbitals are shown in Figure 3 with the corresponding natural orbital occupations. It can be seen that the porphyrin π–π* orbitals are compressed between the V–N and V–O based σ and π bonding orbitals and the corresponding σ* and π* antibonding orbitals. Very importantly, the nonbonding d_*xy*_ orbital, which carries the unpaired electron, is sandwiched between the five porphyrin (π/π*) orbitals and remains localized on the V­(IV) ion.

**Table 3:** Low-Lying Vertical Excitation Spectra and Wave Function Decomposition of the VOTPP Molecule from (13,14) Active Space Calculations Using 10 Doublets and 4 Quartets^a^

	CASSCF wave function (major weightage)			
doublet states	σ_*z* ^2^_σ_*x* ^2^–*y* ^2^_π_*xz*_π_*yz*_π(*p*)π(*p*)d_*xy*_π*(*p*)π*(*p*)π*(*p*)π_xz_^*^π_yz_^*^σ_*x* ^2^–*y* ^2^_^*^σ_*z* ^2^_^*^	NEVPT2 (CASSCF) energy (eV)	bands (NEVPT2 osc. str.)	exp. (eV)
GS	2 2 2 2 2 2 u 0 0 0 0 0 0 0 (83%)	0.0		
first ES	2 2 2 2 u 2 u 0 d 0 0 0 0 0 (83%)	1.76 (2.94)	Q(10^–7^)	1.94–1.97
second ES	2 2 2 2 u 2 u d 0 0 0 0 0 0 (83%)	1.76 (2.94)	*Q*(10^–7^)	
third ES	2 2 2 2 2 u u d 0 0 0 0 0 0 (84%)	2.07 (2.45)	Q(10^–7^)	2.14–2.18
fourth ES	2 2 2 2 2 u u 0 d 0 0 0 0 0 (84%)	2.07 (2.45)	Q(10^–7^)	
fifth ES	2 2 2 2 2 d u u 0 0 0 0 0 0 (34%)	2.14 (3.14)	*Q*(0.02)	2.25–2.30
	2 2 2 2 d 2 u 0 u 0 0 0 0 0 (25%)			
sixth ES	2 2 2 2 2 d u 0 u 0 0 0 0 0 (34%)	2.14 (3.14)	Q(0.02)	
	2 2 2 2 d 2 u u 0 0 0 0 0 0 (25%)			
seventh ES	2 2 2 2 2 2 0 0 0 0 0 0 u 0 (83%)	2.65 (2.40)	-	
eighth ES	2 2 2 2 2 u 0 u 0 0 0 0 d 0 (86%)	4.73 (4.86)	-	
ninth ES	2 2 2 2 2 u 0 0 u 0 0 0 d 0 (86%)	4.73 (4.86)	-	
Quartet States				
first ES	2 2 2 2 u 2 u 0 u 0 0 0 0 0 (83%)	1.76 (2.93)		
second ES	2 2 2 2 u 2 u u 0 0 0 0 0 0 (83%)	1.76 (2.93)		
third ES	2 2 2 2 2 u u u 0 0 0 0 0 0 (84%)	2.07 (2.45)		
fourth ES	2 2 2 2 2 u u 0 u 0 0 0 0 0 (84%)	2.07 (2.45)		

a Only major determinants (u = spin up, d = spin down) are shown; conjugate determinants are omitted.

**Figure 3:**
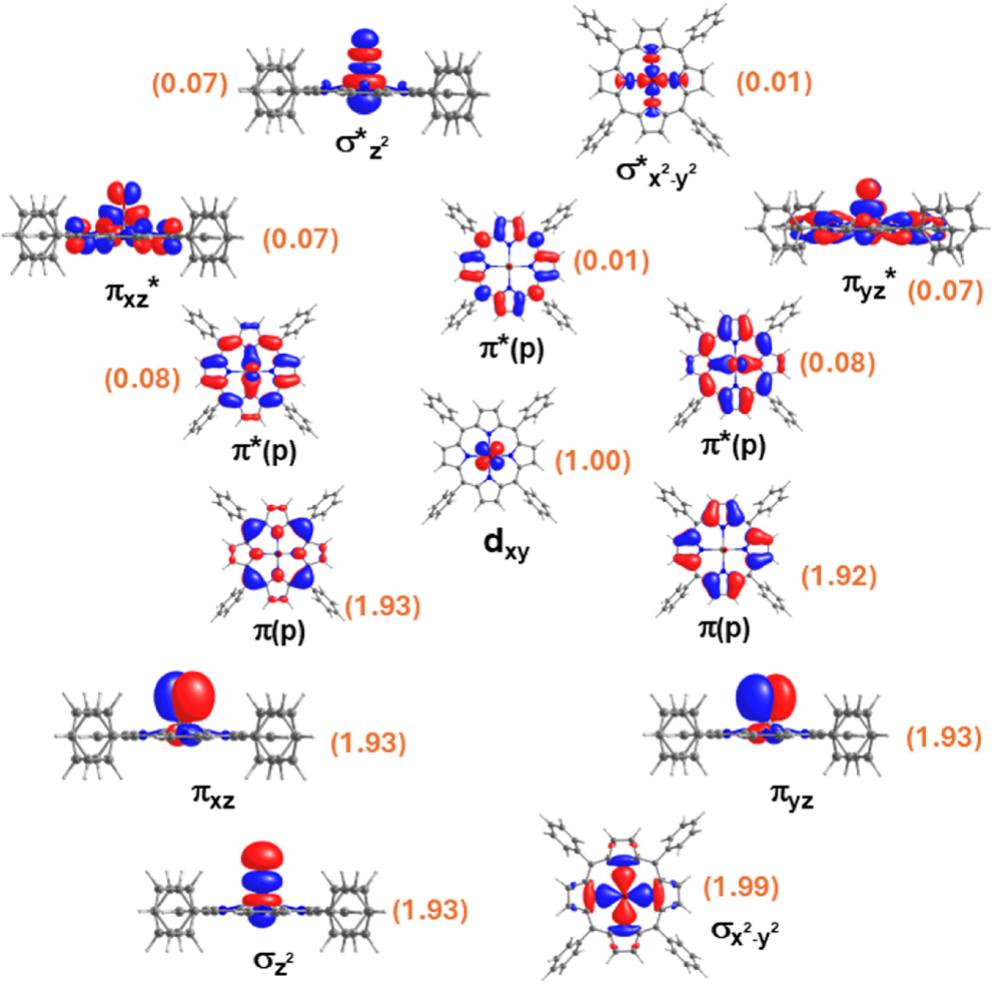
Natural orbitals of VOTPP obtained from state-specific CASSCF­(13,14) calculations along with their natural orbital occupation numbers.

With these sets of active orbitals, SA-CASSCF calculations were carried out using nine excited doublets and four excited quartet states to investigate the low-lying excitations. The state-average configurations (in their determinant form) with their corresponding NEVPT2 vertical excitation energies are shown in Table 3. Notably, the quartet states in the VOTPP are now nearly degenerate with the low-lying doublet states; the first two quartets and doublets lie around 1.76 eV, and the third and fourth quartets and doublets lie at 2.07 eV. A closer inspection reveals that the energy splitting between the first two degenerate quartets and doublets is just 6–10 cm^–1^, and the gap between the second degenerate quartets and doublets is 8 cm^–1^, with the quartet pairs always being lower in energy. The significant distinction from this result of the (13,14) active space is that now the low-lying π–π* transitions are clearly visible, which were absent from the (1,5) and (9,9) active spaces. Indeed, the first six excited states from NEVPT2 are the porphyrin π–π* transitions, thereby giving rise to the Q_*x*_ and Q_*y*_ bands. It is apparent from the comparison of H_2_TPP spectra (see Tables 2 and 3) that the incorporation of the vanadyl cation results in a red shift (lowering in energy) of the Q_*x*_ and Q_*y*_ bands, in agreement with the experimental observations.^53^ The SA-CASSCF­(13,14) results, on the other hand, unsurprisingly overestimate the excited state energies (Table S8 in the SI). Also, there is one metal-based d_*xy*_-d*x* ^2^–*y* ^2^ transition, which is expected to lie higher in energy compared to the π–π* transitions is incorrectly identified from the CASSCF calculations (see Tables 3 and S8). Previous photophysical studies on VOTPP and related molecules^13^ support the presence of low-lying quartet states.^54^ The two higher-lying transitions at 4.73 eV correspond to double excitations associated with porphyrin π-π* and metal-centered d_*xy*_-d_*x* ^2^–*y* ^2^_ excitations.

### Copper Tetraphenyl Porphyrin

Next, we perform active space exploration for the CuTPP molecule. As usual, the metal-based (9,5) material is examined first. The d-orbitals occupation and the corresponding SA-CASSCF and NEVPT2 excitation energies are reported in Table S9 and Figure S3 in the SI. Despite being a one-electron (or one hole) system, there is a clear difference between the V­(IV) and Cu­(II) d-orbital splitting within the TPP ligand environment. Ab initio ligand-field analysis (AI-LFT from NEVPT2 energies) revealed that the ligand-field splitting (difference between the lowest and highest d-orbital energies) in the Cu­(II) center is around 2.2 eV, which is more than 2 eV smaller compared to the V­(IV) center (Tables S5 and S8 in the SI). This is expected as vanadium is in a + 4 oxidation state and additionally bonded to a very electronegative oxygen atom, while copper is in a + 2 oxidation state and not bonded to any electronegative species in the axial direction. Importantly, when the second d-shell is incorporated into the active space for CuTPP, the vertical excitation energies of the first five doublets from NEVPT2­(9,10) are reduced by ∼0.4 eV and SA-CASSCF excited states are increased by ∼0.1 eV (see Tables S9 and S10 in the SI). Interestingly, this is contrary to what we observe for VOTPP, where the double shell minutely affects the energies of the five doublet energies (see Tables S5 and S6 in the SI).

An intermediate active space of (11,6) can be constructed for CuTPP by incorporating the doubly occupied σ_*x* ^2^–*y* ^2^_ orbital into the (1,5) active space consisting of the nitrogen coordinating orbitals. State-average calculations using five doublet roots using this active space, in fact, reinstate the (9,5) active space results from both SA-CASSCF and NEVPT2 energies (see Table S11 and Figure S4 in the SI). This can be attributed to the more balanced electron correlation by including the Cu–N bonding (σ_*x* ^2^–*y* ^2^_) and the antibonding σ_*x* ^2^–*y* ^2^_^*^ orbitals compared to the (9,10) active space where the Cu 3d electrons gained additional delocalization.

Analogous to the VOTPP, a large active space of (17,12) was constructed for the CuTPP molecule. This has been achieved by combining the (4,5) active space of the TPP moiety, (9,5) of the Cu­(II) center, and a degenerate pair of doubly occupied porphyrin π orbitals. We detected that the nitrogen p_*z*_ orbitals contained in this degenerate pair of π orbitals weakly interact with the d_*xz*_/d_*yz*_ orbitals of the Cu­(II) center in the CuTPP molecule. Contrary to the VOTPP case, this degenerate π pair of orbitals was unavailable to accommodate for the VOTPP case as the d_*xz*_/d_*yz*_ orbitals of the V­(IV) center were involved in strong bonding interaction with the oxygen atom. Attempts to optimize the active orbitals only for the ground state were unsuccessful; therefore, at first, state-averaged calculations were performed using five roots. This may be attributed to the close-lying five excited states originating from the five d–d excitations, which were found to lie within 14,000 cm^–1^ obtained from SA-CASSCF energies (see Table S12 in the SI). In fact, the five d-orbitals in the CuTPP from the SA(5)-CASSCF (averaging over five states) calculation all possess a natural orbital occupation of 1.8, indicating a strong electron correlation within the five orbitals (Figure S5 in the SI). However, this lacks complete electron correlation and low-lying π–π* transitions, and hence, the SA(5)-CASSCF orbitals were further optimized using twelve doublets and four quartets. The state-average orbitals are plotted in Figure 4 with their state-average pseudo-natural occupations, and the excited state configurations with their NEVPT2 energies are shown in Table 4. Finally, by using a fair amount of state-average roots, NEVPT2 results ascertain that the first four excited states originate from the porphyrin π–π* transitions (see Table 4). Similar to VOTPP, in the case of CuTPP, the first two doublet pairs and the first two quartet pairs are quite close in energy, and the energy difference between the first quartet pair and the first doublet pair is roughly 170 cm^–1^, with quartet pairs being lower in energy. The next two doublet pairs at 2.12–2.13 eV (585 nm) and 2.16 eV show significant oscillator strength from NEVPT2 calculations, and are in close agreement with the experimental absorption spectra,^55–57^ which is detected around 2.00 eV (620 nm) and 2.30 eV (540 nm) respectively. The SA-CASSCF results, on the other hand, predict the d–d excited states to be lower in energy compared with the porphyrin π–π* transitions (see Table S13 in the SI). The π–π* transitions are also overestimated from the SA-CASSCF calculations, which further emphasize the role of second-order perturbative corrections in determining the excited state spectra of these types of molecules.

**Table 4:** Wave Function Decomposition and Vertical Excited States of the CuTPP Molecule from (17,12) Active Space Calculations^a^

	CASSCF wave function (major weightage)			
doublet states	π(*p*)π(*p*)d_*z* ^2^_d_*xz*_d_*yz*_d_*xy*_ π(*p*)π(*p*)d_*x* ^2^–*y* ^2^_π*(*p*)π*(*p*)π*(*p*)	NEVPT2 (CASSCF) energy(eV)	bands (osc. str.)	exp. (eV)
GS	2 2 2 2 2 2 2 2 u 0 0 0 (88%)	0.0		
first ES	2 2 2 2 2 2 u 2 u 0 d 0 (88%)	1.84 (2.70)		
second ES	2 2 2 2 2 2 u 2 u d 0 0 (88%)	1.85 (2.68)		
third ES	2 2 2 2 2 u 2 2 2 0 0 0 (88%)	2.03 (1.46)		
fourth ES	2 2 2 2 2 2 2 u u 0 d 0 (89%)	2.12 (2.35)	Q(10^–6^)	2.00
fifth ES	2 2 2 2 2 2 2 u u d 0 0 (89%)	2.13 (2.32)	Q(10^–6^)	
sixth ES	2 2 2 2 2 2 2 u u d 0 0 (51%)	2.16 (2.98)	Q(0.02)	2.30
	2 2 2 2 2 2 u 2 u 0 d 0 (36%)			
seventh ES	2 2 2 2 2 2 2 u u 0 d 0 (51%)	2.16 (2.96)	Q(0.02)	
	2 2 2 2 2 2 u 2 u d 0 0 (37%)			
eighth ES	2 2 2 2 u 2 2 2 2 0 0 0 (67%)	2.16 (1.72)		
	2 u 2 2 2 2 2 2 2 2 0 0 (21%)			
ninth ES	2 2 2 u 2 2 2 2 2 0 0 0 (67%)	2.17 (1.72)		
	u 2 2 2 2 2 2 2 2 2 0 0 (09%)			
10th ES	2 2 u 2 2 2 2 2 2 0 0 0 (88%)	2.27 (1.81)		
11th ES	2 2 2 2 2 u 2 u 2 d 0 0 (89%)	4.15 (3.77)		
Quartet States				
first ES	2 2 2 2 2 2 u 2 u u 0 0 (88%)	1.82 (2.67)		
second ES	2 2 2 2 2 2 u 2 u 0 u 0 (88%)	1.83 (2.67)		
third ES	2 2 2 2 2 2 2 u u 0 u 0 (89%)	2.11 (2.34)		
fourth ES	2 2 2 2 2 2 2 u u u 0 0 (89%)	2.12 (2.31)		

a Only major determinants (u = spin up, d = spin down) are shown, conjugate determinants are omitted.

**Figure 4:**
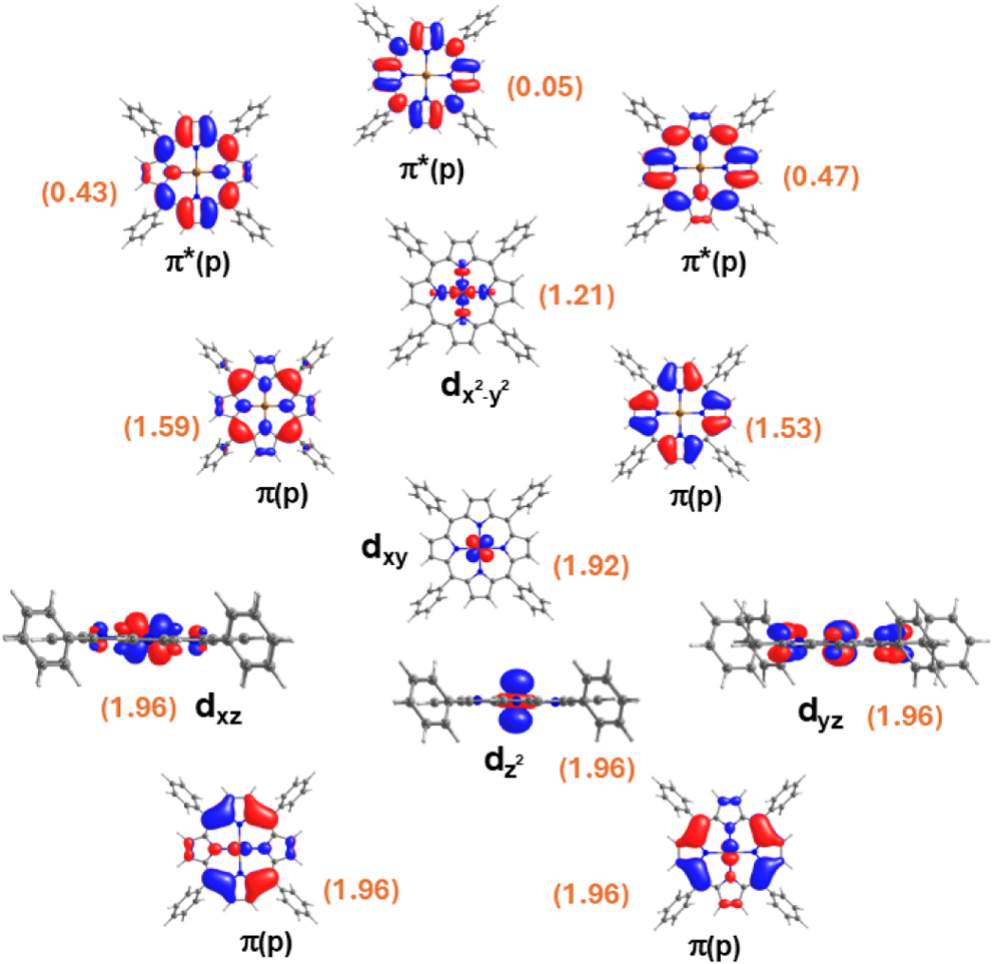
Active orbitals of CuTPP obtained from SA-CASSCF­(17,12) calculations using 12 doublets and 4 quartets. The numbers in orange correspond to their pseudonatural orbital occupation numbers.

Figure 5 enables a comparative analysis of their relative band positions and excitation characteristics, with NEVPT2 transition energies from selected active spaces providing insight into the correlation between band positions, excitation energies, and nature of the underlying electronic transitions. In all of these multireference calculations, Q-bands were obtained as the first or lowest-lying intense band associated with the porphyrin ring and can be achieved using most of the active spaces, except where the active spaces exclude the delocalized porphyrin orbitals. The Soret band can only be located if the active space size is at least or greater than (8,7) with a reasonable number of roots. This observation was also reported earlier from CASPT2 calculations in free base porphyrin.^37^ Ligand-to-metal charge transfer (LMCT) states associated with the vanadyl moiety are situated quite far, around 5.5 eV, and are very high-energy bands. Doubles excitations manifested by d–d and π–π* transitions appear between 4 and 5 eV, both for VOTPP and CuTPP molecules when large active spaces are used. While in the case of VOTPP, the d-d transitions are quite separated from the π–π* Q-bands, the CuTPP d-d transitions coincide with the Q-band transitions.

**Figure 5:**
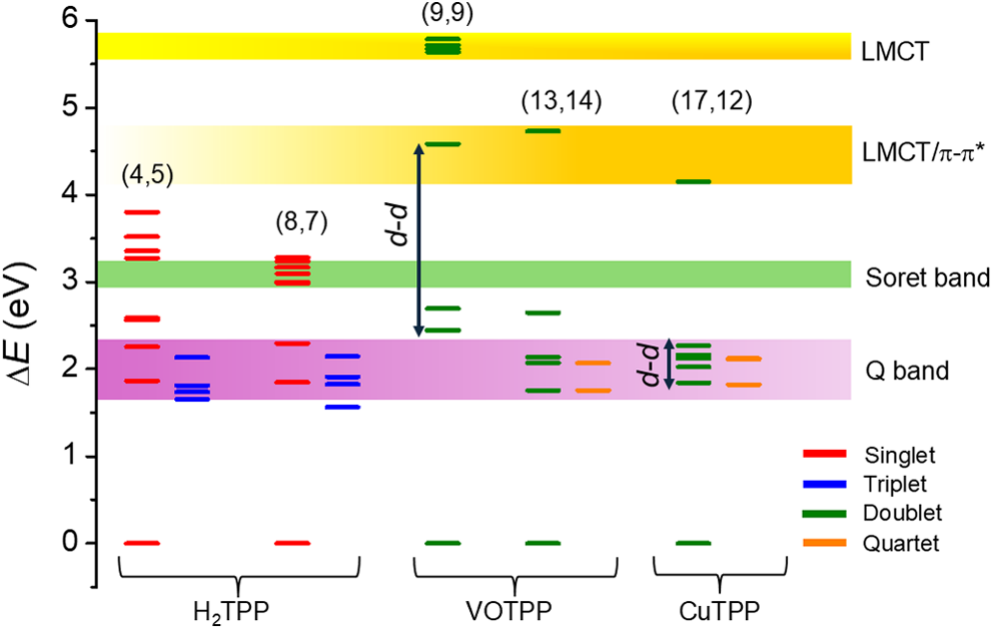
Comparison of the excited states from various active spaces of the three molecules and their respective band positions obtained from NEVPT2 calculations.

### Spin–Orbit Coupling and *g*-Shifts

For all of the active spaces discussed above for VOTPP and CuTPP, *g*-tensors are computed using QDPT (SOC) followed by the effective Hamiltonian approach (EHA) for the ground state Kramers pair (see Table 5). The largest contribution to the *g*-value in the case of transition metal complexes is from the spin–orbit/Zeeman interaction, which is represented within a perturbation theory approach by^29^

1 $$g_{KL}^{\left(\mathrm{OZ}/\mathrm{SOC}\right)}=-\frac{1}{S}\sum_{b\left(S_{b}=S\right)}\Delta_{b}^{-1}\left\{\left\langle \Psi_{0}^{SS}\left|\sum_{i}l_{i;K}\right|\Psi_{b}^{SS}\right\rangle \left\langle \Psi_{b}^{SS}\left|\sum_{i}z_{L;i}s_{z;i}\right|\Psi_{0}^{SS}\right\rangle +\left\langle \Psi_{0}^{SS}\left|\sum_{i}z_{K;i}s_{z;i}\right|\Psi_{b}^{SS}\right\rangle \left\langle \Psi_{b}^{SS}\left|\sum_{i}l_{i;L}\right|\Psi_{0}^{SS}\right\rangle \right\}$$

**Table 5:** Comparison of the Calculated and Experimental g-Factors of the VOTPP and CuTPP Molecules^a^

species	method/number of excited states	*g*_*x*_	*g*_*y*_	*g*_*z*_
VOTPP	SA-CASSCF(1,5)	1.984	1.984	1.934
	5 doublets			
	NEVPT2(1,5)	1.988	1.988	1.947
	5 doublets			
	SA-CASSCF(1,10)	1.984	1.984	1.934
	5 doublets			
	NEVPT2(1,10)	1.988	1.988	1.946
	5 doublets			
	SA-CASSCF(9,9)	1.989	1.989	1.939
	5 doublets			
	NEVPT2(9,9)	1.986	1.986	1.946
	5 doublets			
	SA-CASSCF(9,9)	1.988	1.988	1.940
	9 doublets			
	NEVPT2(9,9)	1.987	1.987	1.948
	9 doublets			
	SA-CASSCF(13,14)	2.002	2.002	1.938
	10 doublets and 4 quartets			
	NEVPT2(13,14)	2.002	2.002	1.944
	10 doublets and 4 quartets			
exp		1.986	1.986	1.963
CuTPP	SA-CASSCF(9,5)	2.097	2.098	2.501
	5 doublets			
	NEVPT2(9,5)	2.078	2.078	2.347
	5 doublets			
	SA-CASSCF(9,10)	2.097	2.097	2.491
	5 doublets			
	NEVPT2(9,10)	2.093	2.094	2.446
	5 doublets			
	SA-CASSCF(11,6)	2.098	2.098	2.502
	5 doublets			
	NEVPT2(11,6)	2.079	2.079	2.351
	5 doublets			
	SA-CASSCF(17,12)	2.097	2.098	2.500
	5 doublets			
	NEVPT2(17,12)	2.078	2.078	2.347
	5 doublets			
	SA-CASSCF(17,12)	2.089	2.089	2.478
	12 doublets and 4 quartets			
	NEVPT2(17,12)	2.076	2.076	2.349
	12 doublets and 4 quartets			
exp		2.065	2.065	2.200

a Experimental values were taken from ref 10 for VOTPP and ref 7 for CuTPP.

Here, *S* represents the ground state spin of the species, 1/2 in our case, Ψ_0_^SS^ and Ψ_*b*_^SS^ represent the ground state wave function and the excited state wave function within the same *S* multiplet. *l*_*i;K*_ and *l*_*i;L*_ are the *K*th and *L*th components of the orbital angular momentum of the electron. Here, *z*_*L;i*_ is the *L*th component of the effective one-electron bond obtained from the SOMF approximation to the full SOC operator. For the vanadyl, *g*_*z*_ is expected to be small compared to *g*_*x*_ and *g*_*y*_ as the numerator of eq 1 is larger for transition d_*xy*_-d_*x* ^2^–*y* ^2^_ compared to d_*xy*_-d_*xz*_/d_*yz*_ transitions. Except for the large (13,14) active space of VOTPP, the other active spaces include all five d–d excitations, which are essentially required to perturb the three *g*-factors from the free electron value of 2.0023. For the (13,14) active space, ten doublet roots are not enough to incorporate all the d–d excitations except the d_*xy*_-d_*x* ^2^–*y* ^2^_ transition, and hence, both the *g*_*x*_ and *g*_*y*_ values for this active space are close to 2.0023. Among the results of NEVPT2 (1,5) and NEVPT2 (1,10), no significant differences are observed in the *g* factors. Inclusion of the LMCT states, that is, in the (9,9) active space, significantly improves the *g*-factors in comparison to experimental (EPR) values, particularly for *g*_*x*_ and *g*_*y*_.

Due to the presence of pseudo-*D*_4*h*_ symmetry, the *g*_*x*_ and *g*_*y*_ components are similar for both molecules and are usually referred to as *g*_⊥_. In the case of CuTPP, *g*_*z*_ is larger than the *g*_*x*_ and *g*_*y*_ values as the relative splitting between the d_*xy*_-d*x* ^2^–*y* ^2^ orbitals is smaller in the case of CuTPP than from VOTPP. Here, a clear difference is visible between the NEVPT2­(9,5) and NEVPT2­(9,10) *g*-factors, since the spin–orbit free excitation energies were underestimated in the (9,10) active space from NEVPT2­(9,10) method, which significantly overestimates the computed *g*-factors. This observation has previously been encountered in the estimation of *g*-factors using NEVPT2­(9,10) methods.^29^ The (11,6) active space, on the other hand, outperforms the (9,10) active space in this regard, providing reasonable *g*-factors for the CuTPP molecule. Unlike VOTPP, in the case of CuTPP, the largest active space (17,12) with 12 doublets includes all four d–d excited states and hence exhibits satisfactory *g*-factors compared to the other active space results. In fact, the SA-CASSCF­(17,12) and NEVPT2­(17,12) computed *g*-factors are comparable to those of (9,5) active space ones.

## Discussion and Conclusions

Four different active spaces have been chosen to inspect the ligand-to-metal charge transfer (LMCT), ligand-centric π–π* and metal-centric d-d excitations in VOTPP and CuTPP molecular qubits. For the free H_2_TPP molecule, the (8,7) active space captures all the required Q_*x*_/Q_*y*_ and Soret bands in order to explain the experimental absorption spectra compared to the smaller (4,5) active space, which is insufficient to determine the high-intensity Soret bands. For VOTPP and CuTPP, the minimal ((1,5) or (9,5)) and the double-shell embedded ((1,10) or (9,10)) active spaces primarily account for the d–d excitations but do not provide a full description of the complete spin state energies and do not align with the experimental absorption spectra. However, these active spaces yield a very accurate estimation of the *g*-factors. Intermediate active spaces such as (9,9) for VOTPP or (11,6) for CuTPP, which account for the metal–ligand bonding interaction up to the first coordination shell, improve the *g*-values to some extent but are insufficient to reproduce the low-lying π–π* transitions. Largest active space models such as (13,14) and (17,12), on the other hand, quantitatively describe the low-lying π–π* excited states efficiently for both molecules. Largest active space calculations confirm that both for the VOTPP and CuTPP molecules, the low-lying excited states are dominated by quartet states.

This last observation, in particular, has potentially strong repercussions on our current understanding of spin relaxation and decoherence in this prominent class of molecular spin qubits. Indeed, the energy of the low-lying excited states in a molecular complex has been found to be key in determining the rate of Raman relaxation.^24^ The latter becomes the leading dissipation mechanism above about 20 K and limits coherence times in molecules at room temperature, and its mitigation is therefore of paramount importance for advancing molecular complexes in the field of quantum information science. So far, the community has been focusing on increasing the crystal field of metal ions to increase the separation between ground and excited states.^21,58^ However, this approach accounts for only d–d excitations and neglects the possibility of other electronic excitations to play a role in the relaxation process. In this regard, our study provides novel insights into the reciprocal positions of various electronic excitations. Importantly, it shows that VOTPP’s d–d excitations are very high in energy and that ligand-based electronic transitions are determining the low-lying electronic spectrum. In the case of CuTPP, much smaller crystal field splittings are observed, making d-d transitions a relevant feature of the low-lying excitations but still strongly admixed to the π–π* transitions of the TPP ligand. The recent development of a theory of relativistic vibronic coupling^59^ and spin relaxation theory based on the full electronic structure of molecular compounds^24^ will make it possible to include such electronic features into spin dynamics to fully elucidate their role, and work in this direction is ongoing. These observations are also strongly relevant for the emerging field of the optical control of magnetic molecules. While several strategies are being pursued to achieve initialization and read-out of ground state spins through optical means, the possibility to control luminescence and radiative decays from low-lying excited states is a common denominator. While a general theory for such processes in open-shell compounds is yet to be achieved, the current results represent a first step in that direction. In this regard, the observation that VOTPP possesses very high-energy d–d states and low-lying π–π* ones points to this system as very promising to achieve ligand-based emission after d-d excitation, which possibly opens up ways to read-out magnetic states.

In conclusion, our study thus makes an important step in correctly representing the low-lying excited states of this central class of molecular qubits and paves the way to an investigation of the role of these overlooked electronic features in the spin–lattice relaxations and photophysical properties of spin-1/2 molecules.

## Supplementary Material



## Notes

Wave function decomposition and excited state energies at the SA-CASSCF and NEVPT2 level using (8,7) active space (Tables S1–S2) and with (4,5) active space for H_2_TPP (Table S3), with (8,7) active space for TPP dianion (Table S4); wave function decomposition and vertical excitations of VOTPP using (1,5), (1,10), (9,9), and (13,14) active spaces (Tables S5–S8); VOTPP (1,5) and (9,9) active orbitals (Figures S1 and S2); wave function decomposition and vertical excitations for CuTPP are reported using (9,5), (9,10), (11,6), (17,12) active spaces (Tables S9–S13); canonical/natural orbitals using (9,5), (11,6), (17,12) active spaces for CuTPP molecule (Figures S3–S5); and coordinates are given in the *xyz* format of the gas-phase optimized geometries (PDF)
