# Supplementary material for: A Multireference Picture of Electronic Excited States in Vanadyl and Copper Tetraphenyl Porphyrin Molecular Qubits
Source: J Phys Chem A. 2025 Jul 29;129(31):7160–9. doi: 10.1021/acs.jpca.5c03946 (PMC12337143; doi:10.1021/acs.jpca.5c03946)
Supplement: Supplementary file 1 [file jp5c03946_si_001.pdf]

Supporting Information

A Multireference Picture of Electronic Excited States in Vanadyl and Copper Tetraphenyl Porphyrin Molecular Qubits

Arup Sarkar,* Alessandro Lunghi*

School of Physics, AMBER and CRANN Institute, Trinity College, Dublin 2, Ireland

Email: lunghia@tcd.ie, arsarkar@tcd.ie

Contents:

	Page no.
Table S1. Wave function decomposition and vertical excited states for the H_2TPP from NEVPT2(8,7) calculations	S2
Table S2. Wave function decomposition and vertical excited states for the H_2TPP from SA-CASSCF(8,7) calculations	S3
Table S3: Wave function decomposition and excited states for the H_2TPP from SA-CASSCF/NEVPT2(4,5) calculations	S3
Table S4: Excited state analysis for the TPP dianion from SA-CASSCF(8,7)/NEVPT2 calculations	S4
Table S5: Vertical excited states obtained from the (1,5) active space for the VOTPP molecule.	S5
Table S6: Vertical excited states obtained from the (1,10) active space for the VOTPP molecule.	S5
Figure S1. Ab initio ligand field (NEVPT2) orbitals from the (1,5) active space of the VOTPP molecule.	S6
Table S7: Vertical excited states and wave function from the (9,9) active space for the VOTPP molecule	S6
Figure S2. State-average (canonical) orbitals from CAS(9,9) active space of VOTPP molecule.	S7
Table S8: Vertical excited states from the SA-CASSCF(13,14) calculations for the VOTPP molecule.	S8
Table S9: Vertical excited states obtained from the (9,5) active space for the CuTPP molecule.	S9
Table S10: Vertical excited states obtained from the (9,10) active space for the CuTPP molecule.	S9
Figure S3. Ab initio ligand-field (NEVPT2) orbitals from CAS(9,5) active space of CuTPP molecule.	S10
Table S11: Vertical excited states from the (11,6) active space calculations for the CuTPP molecule.	S10
Figure S4. Pseudo natural orbitals from SA(5)-CASSCF(11,6) active space of the CuTPP molecule.	S11
Table S12: Vertical excited states obtained from the SA-CASSCF(17,12) calculations for the CuTPP molecule using 5 roots	S11
Figure S5. State-average (5 roots) canonical orbitals from SA-CASSCF(17,12) active space of CuTPP molecule.	S12

Table S13: Vertical excited states obtained from the SA-CASSCF(17,12) calculations for the CuTPP molecule using 12 doublets and 4 quartets S12
Coordinates (xyz format) S13

Table S1. Wave function decomposition and vertical excited states for the H_2TPP from NEVPT2(8,7) calculations using ten triplet and ten singlet roots. Here results are shown up to the 6th excited singlet and 4th excited triplet. The electronic occupancy of the CASSCF wave functions is shown with respect to the orbitals shown in the main manuscript in Figure 2. Only dominant configurations (determinants, *u*=up, *d*=down spin) are shown; the conjugate determinants are omitted for clarity.

Singlet states	CASSCF wave function (major weightage)	NEVPT2 excitation energy (eV)	Bands (Osc. Str.)	Exp (eV)
GS	2 2 2 2 0 0 0 (91%)	0.0		
1st ES	2 2 u 2 0 d 0 (49%)<br>2 2 2 u d 0 0 (43%)	1.85	Q_x (0.02)	1.91-2.07
2nd ES	2 2 2 u 0 d 0 (49%)<br>2 2 u 2 d 0 0 (44%)	2.29	Q_y (0.01)	2.25-2.42
3rd ES	u 2 2 2 d 0 0 (29%)<br>2 2 u 2 d 0 0 (28%)<br>2 2 2 u 0 d 0 (25%)	2.98	Soret (1.46)	3.10-3.33
4th ES	u 2 2 2 0 d 0 (49%)<br>2 2 2 u d 0 0 (19%)	3.00	Soret (0.2)	
5th ES	2 u 2 2 0 d 0 (78%)	3.10	-	
6th ES	u 2 2 2 d 0 0 (46%)<br>2 2 u 2 d 0 0 (18%)	3.17	Soret (0.2)	
7th ES	2 u 2 2 d 0 0 (71%)	3.24	-	
8th ES	2 2 u 2 0 0 d (46%)<br>2 2 u d 2 0 0 (20%)	3.28	-	
Triplet states				
1st ES	2 2 u 2 u 0 0 (58%)<br>2 2 2 u 0 u 0 (35%)	1.57		
2nd ES	2 2 u 2 0 u 0 (87%)	1.83		
3rd ES	2 2 2 u 0 u 0 (60%)<br>2 2 u 2 u 0 0 (34%)	1.91		
4th ES	2 2 2 u u 0 0 (90%)	2.15		

Table S2. Wave function decomposition and vertical excited states for the H_2TPP from SA-CASSCF(8,7) calculations using ten triplets and ten singlets. Results are shown up to the 8th excited singlet and 4th excited triplet. The electronic occupancy of the CASSCF wave functions is shown with respect to the orbitals shown in the main manuscript in Figure 2. Only dominant configurations (determinants, *u*=up, *d*=down spin) are shown; the conjugate determinants are omitted for clarity.

Singlet states	CASSCF wave function (major weightage)	CASSCF excitation energy (eV)	Bands (Osc. Str.)	Exp (eV)
GS	2 2 2 2 0 0 0 (91%)	0.0		
1st ES	2 2 u 2 0 d 0 (49%)<br>2 2 2 u d 0 0 (43%)	2.88	Q_x (0.03)	1.91-2.07
2nd ES	2 2 2 u 0 d 0 (49%)<br>2 2 u 2 d 0 0 (44%)	3.20	Q_y (0.013)	2.25-2.42
3rd ES	2 u 2 2 0 d 0 (78%)	4.19	-	
4th ES	u 2 2 2 0 d 0 (49%)<br>2 2 2 u d 0 0 (19%)	4.22	Soret (0.3)	3.10-3.33
5th ES	2 u 2 2 d 0 0 (71%)	4.47	-	
6th ES	u 2 2 2 d 0 0 (46%)<br>2 2 u 2 d 0 0 (18%)	4.51	Soret (0.34)	
7th ES	2 2 2 u 0 0 d (48%)<br>2 2 2 0 2 0 0 (22%)	5.00	-	
8th ES	u 2 2 2 d 0 0 (29%)<br>2 2 u 2 d 0 0 (28%)<br>2 2 2 u 0 d 0 (25%)	5.15	Soret (2.51)	
Triplet states				
1st ES	2 2 u 2 u 0 0 (58%)<br>2 2 2 u 0 u 0 (35%)	2.42		
2nd ES	2 2 2 u u 0 0 (90%)	2.46		
3rd ES	2 2 2 u 0 u 0 (60%)<br>2 2 u 2 u 0 0 (34%)	2.61		
4th ES	2 2 u 2 0 u 0 (87%)	2.85		

Table S3: Wave function decomposition and excited states for the H_2TPP from SA-CASSCF/NEVPT2(4,5) calculations using 10 triplets and 10 singlets. Results are shown up to the 8th excited singlet and 4th excited triplet. Only dominant configurations (determinants, *u*=up, *d*=down spin) are shown; the conjugate determinants are omitted for clarity.

Singlet states	CASSCF wave function $\pi(p)\pi(p)\pi^*(p)\pi^*(p)\pi^*(p)$	NEVPT2 (CASSCF) excitation energy (eV)	Bands (NEVPT2 Osc. Str.)	Exp (eV)
GS	2 2 0 0 0 (92%)	0.0		
1st ES	2 u d 0 0 (49%)<br>u 2 0 d 0 (45%)	1.87 (2.92)	Q_x (0.01)	1.91-2.07
2nd ES	2 u 0 d 0 (52%)<br>u 2 d 0 0 (43%)	2.26 (3.19)	Q_y (0.01)	2.25-2.42

3rd ES	2 u 0 0 d (52%)<br>2 0 0 2 0 (21%)	3.80 (4.96)	-	Soret (3.10-3.33)
4th ES	u 2 0 d 0 (46%)<br>2 u d 0 0 (41%)	2.56 (4.98)	Shoulder (1.54)	
5th ES	u 2 d 0 0 (48%)<br>2 u 0 d 0 (40%)	2.59 (5.02)	Shoulder (1.44)	
6th ES	u 2 0 0 d (43%)<br>u d 2 0 0 (20%)	3.27 (5.24)	-	
7th ES	u d u d 0 (60%)	3.52 (5.67)	-	
8th ES	0 2 u d 0 (43%)<br>2 0 u d 0 (18%)	3.35 (5.77)	-	
Triplet states				
1st ES	2 u 0 u 0 (82%)	1.81 (2.39)		
2nd ES	2 u u 0 0 (94%)	2.13 (2.51)		
3rd ES	u 2 u 0 0 (82%)	1.65 (2.64)		
4th ES	u 2 0 u 0 (93%)	1.74 (2.85)		

Table S4: Excited state analysis for the TPP dianion from SA-CASSCF(8,7)/NEVPT2 calculations using ten triplet and ten singlet roots. Results are shown up to the 8th excited singlet and 4th excited triplet. Only dominant configurations (determinants, *u*=up, *d*=down spin) are shown; the conjugate determinants are omitted for clarity.

Singlet states	CASSCF wave function<br>π(p)π(p)π(p)π(p)π*(p)π*(p)π*(p)	CASSCF excitation energy (eV)	NEVPT2 Transition energies (eV)
GS	2 2 2 2 0 0 0 (87%)	0.0	0.0
1st ES	2 2 2 u 0 d 0 (44%)<br>2 2 u 2 d 0 0 (37%)	2.52 (Q_x/Q_y)	1.92 (Q_x/Q_y)
2nd ES	2 2 2 u d 0 0 (44%)<br>2 2 u 2 0 d 0 (38%)	2.61 (Q_x/Q_y)	1.87(Q_x/Q_y)
3rd ES	2 2 2 u 0 0 d (34%)<br>2 2 2 0 2 0 0 (17%)<br>2 2 u d u d 0 (13%)	3.69	2.89
4th ES	2 2 u 2 0 0 d (35%)<br>2 2 0 2 u d 0 (19%)	3.91	3.47
5th ES	2 2 u 2 d 0 0 (38%)<br>2 2 2 u 0 d 0 (30%)	4.31 (Soret)	2.54 (Soret)
6th ES	2 2 u 2 0 d 0 (36%)<br>2 2 2 u d 0 0 (20%)	4.32 (Soret)	2.54 (Soret)
7th ES	2 2 2 0 u d 0 (27%)<br>2 2 u d 2 0 0 (24%)	4.65	3.29
8th ES	2 2 u d u d 0 (43%)<br>2 2 2 0 2 0 0 (17%)	4.73	3.37
Triplet states			
1st ES	2 2 2 u u 0 0 (85%)	1.94	1.46

2nd ES	2 2 2 u 0 u 0 (82%)	1.99	1.48
3rd ES	2 2 u 2 0 u 0 (84%)	2.23	2.02
4th ES	2 2 u 2 u 0 0 (82%)	2.27	2.07

Table S5: Vertical excited states obtained from the (1,5) active space for the VOTPP molecule.

Excited states	AI-LFT wave function	SA-CASSCF energy (eV)	NEVPT2 energy (eV)
GS	$d_{xy}^1d_{xz}d_{yz}d_{x^2-y^2}d_{z^2}$	0.0	0.0
1st ES	$d_{xy}d_{xz}^1d_{yz}d_{x^2-y^2}d_{z^2}$	2.14	2.83
2nd ES	$d_{xy}d_{xz}d_{yz}^1d_{x^2-y^2}d_{z^2}$	2.14	2.84
3rd ES	$d_{xy}d_{xz}d_{yz}d_{x^2-y^2}^1d_{z^2}$	2.29	2.84
4th ES	$d_{xy}d_{xz}d_{yz}d_{x^2-y^2}d_{z^2}^1$	4.00	4.57

Table S6: Vertical excited states obtained from the (1,10) active space for the VOTPP molecule.

Excited states	SA-CASSCF energy (eV)	NEVPT2 energy (eV)
GS	0.0	0.0
1st ES	2.14	2.79
2nd ES	2.14	2.83
3rd ES	2.29	2.83
4th ES	4.00	4.57

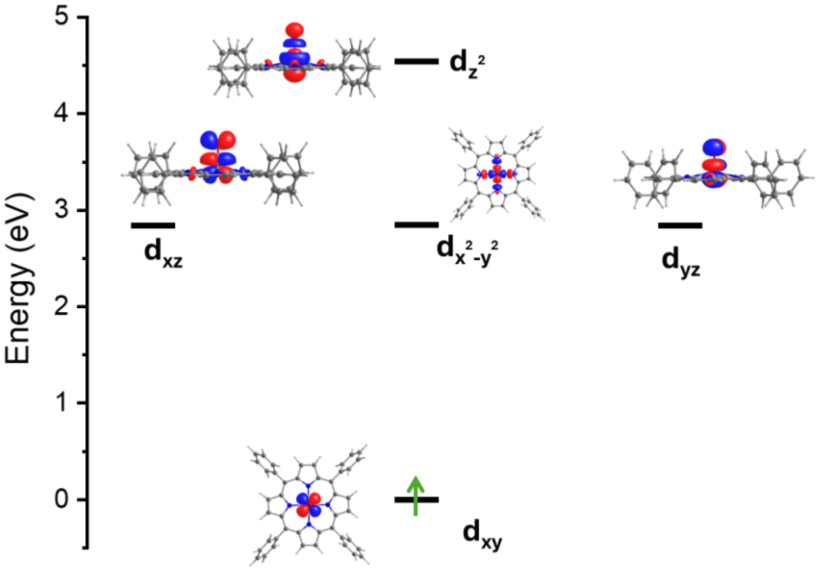

Figure S1. Ab initio ligand field (NEVPT2) orbitals from (1,5) active space of VOTPP molecule. The electronic occupation corresponds to the dominant ground state configuration.

Table S7: Vertical excited states and wave function from the (9,9) active space using 9 doublets for the VOTPP molecule. Only dominant configurations (determinants, *u*=up, *d*=down spin) are shown; the conjugate determinants are omitted for clarity.

Excited states	CASSCF Wave function (major weightage) $\sigma_{z^2}\sigma_{x^2-y^2}\pi_{xz}\pi_{yz}d_{xy}d_{xz}d_{yz}d_{x^2-y^2}d_{z^2}$	SA-CASSCF energy (eV)	NEVPT2 energy (eV)
GS	2 2 2 2 u 0 0 0 0 (84%)	0.0	0.0
1st ES	2 2 2 2 0 0 0 u 0 (84%)	2.37	2.70
2nd ES	2 2 2 2 0 u 0 0 0 (77%)	2.74	2.45
3rd ES	2 2 2 2 0 0 u 0 0 (77%)	2.74	2.45
4th ES	2 2 2 2 0 0 0 0 u (58%)	5.20	4.58
5th ES	2 2 u 2 u d 0 0 0 (44%)<br>2 2 2 u u 0 d 0 0 (44%)	5.73	5.67
6th ES	2 2 2 u u d 0 0 0 (45%)<br>2 2 u 2 u 0 d 0 0 (44%)	5.81	5.71

7th ES	2 2 u 2 u 0 u 0 0 (33%)<br>2 2 2 u u d 0 0 0 (32%)	5.93	5.63
8th ES	2 2 2 u u 0 d 0 0 (45%)<br>2 2 u 2 u d 0 0 0 (44%)	6.03	5.79

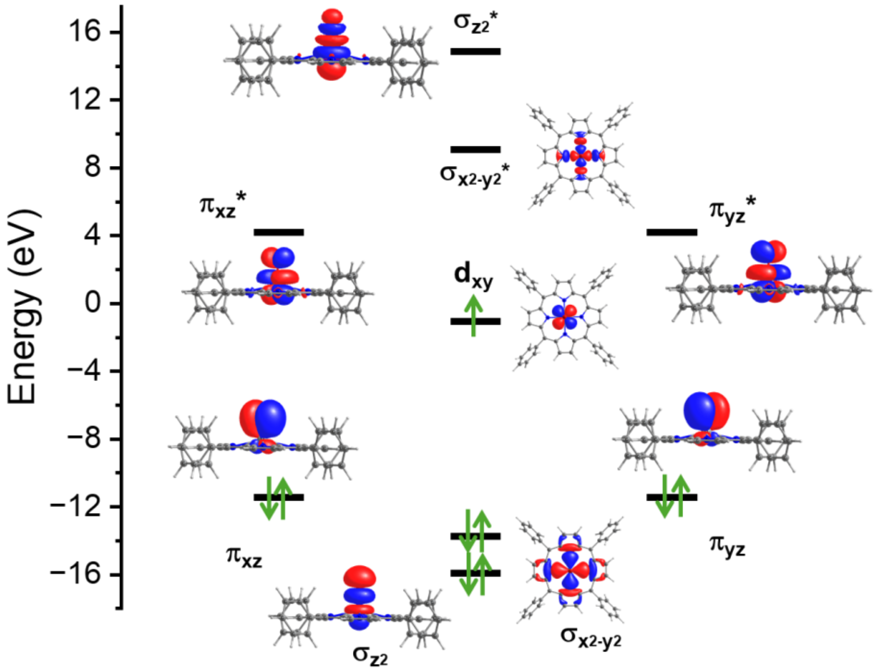

Figure S2. State-average (canonical) orbitals from CAS(9,9) active space of VOTPP molecule. The electronic occupation corresponds to the dominant ground state configuration.

Table S8: Vertical excited states from the SA-CASSCF(13,14) calculations using 10 doublets and 4 quartets for the VOTPP molecule. Only dominant configurations (determinants, *u*=up, *d*=down spin) are shown; the conjugate determinants are omitted for clarity.

Excited states	CASSCF Wave function (major weightage)<br>$\sigma_{z^2}\sigma_{x^2-y^2}\ \pi_{xz}\pi_{yz}\ \pi(p)\pi(p)d_{xy}\pi(p)^*\pi(p)^*\ \pi(p)^*\ \pi_{xz}^*\pi_{yz}^*\ \sigma^*_{x^2-y^2}\sigma^*_{z^2}$	SA-CASSCF energy (eV)	Osc. Str.
GS	2 2 2 2 2 2 u 0 0 0 0 0 0 0 (83%)	0.0	
1st ES	2 2 2 2 2 2 0 0 0 0 0 0 u 0 (83%)	2.40	-
2nd ES	2 2 2 2 2 u u 0 d 0 0 0 0 0 (84%)	2.45	Q(10^{-6})
3rd ES	2 2 2 2 2 u u d 0 0 0 0 0 0 (84%)	2.45	Q(10^{-6})
4th ES	2 2 2 2 u 2 u 0 d 0 0 0 0 0 (83%)	2.94	Q(10^{-7})
5th ES	2 2 2 2 u 2 u d 0 0 0 0 0 0 (83%)	2.94	Q(10^{-7})
6th ES	2 2 2 2 2 d u u 0 0 0 0 0 0 (34%)<br>2 2 2 2 d 2 u 0 u 0 0 0 0 0 (25%)	3.14	Q(0.03)
7th ES	2 2 2 2 2 d u 0 u 0 0 0 0 0 (34%)<br>2 2 2 2 d 2 u u 0 0 0 0 0 0 (25%)	3.14	Q(0.03)
8th ES	2 2 2 2 2 u 0 0 u 0 0 0 d 0 (86%)	4.86	-
9th ES	2 2 2 2 2 u 0 u 0 0 0 0 d 0 (86%)	4.86	-
Quartet states			
1st ES	2 2 2 2 2 u u 0 u 0 0 0 0 0 (84%)	2.45	-
2nd ES	2 2 2 2 2 u u u 0 0 0 0 0 0 (84%)	2.45	-
3rd ES	2 2 2 2 u 2 u 0 u 0 0 0 0 0 (83%)	2.93	
4th ES	2 2 2 2 u 2 u u 0 0 0 0 0 0 (83%)	2.93	

Table S9: Vertical excited states obtained from the (9,5) active space using 5 doublets for the CuTPP molecule.

Excited states	Major CASSCF wave function d_{z^2} d_{xz} d_{yz} d_{xy} $d_{x^2-y^2}$	SA-CASSCF energy (eV)	NEVPT2 energy (eV)
GS	2 2 2 2 1	0.0	0.0
1st ES	2 2 2 1 2	1.37	2.02
2nd ES	2 2 1 2 2	1.56	2.13
3rd ES	2 1 2 2 2	1.56	2.13
4th ES	1 2 2 2 2	1.63	2.22

Table S10: Vertical excited states obtained from the (9,10) active space using 5 doublets for the CuTPP molecule.

Excited states	Major CASSCF wave function d_{z^2} d_{xz} d_{yz} d_{xy} $d_{x^2-y^2}$	SA-CASSCF energy (eV)	NEVPT2 energy (eV)
GS	2 2 2 2 1	0.0	0.0
1st ES	2 2 2 1 2	1.44	1.60
2nd ES	2 2 1 2 2	1.65	1.76
3rd ES	2 1 2 2 2	1.65	1.76
4th ES	1 2 2 2 2	1.74	1.85

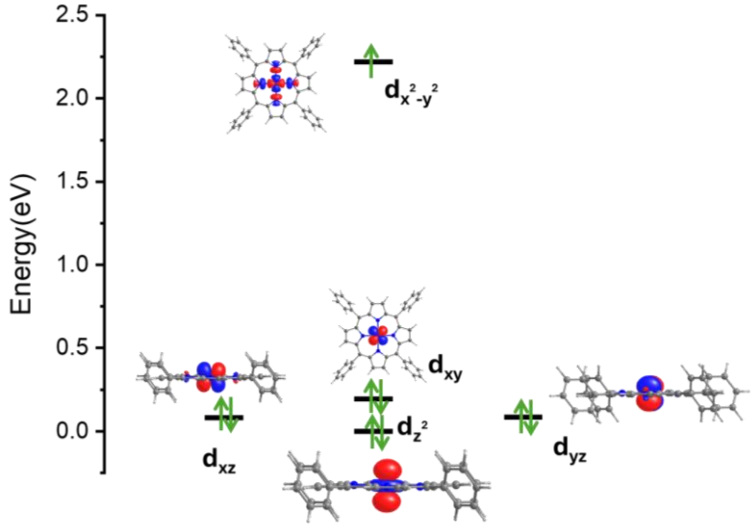

Figure S3. Ab initio ligand-field (NEVPT2) orbitals from CAS(9,5) active space of CuTPP molecule. The electronic occupation corresponds to the CASSCF dominant ground state configuration.

Table S11: Vertical excited states from the (11,6) active space calculations using 5 doublets for the CuTPP molecule.

Excited states	CASSCF wave function (weightage) $\sigma_{x^2-y^2} d_{z^2} d_{xz} d_{yz} d_{xy} d^*_{x^2-y^2}$	SA-CASSCF energy (eV)	NEVPT2 energy (eV)
GS	2 2 2 2 2 1 (62%)	0.0	0.0
1st ES	2 2 2 2 1 2 (94%)	1.37	2.00
2nd ES	2 2 2 1 2 2 (94%)	1.56	2.10
3rd ES	2 2 1 2 2 2 (99%)	1.56	2.10
4th ES	2 1 2 2 2 2 (63%)	1.63	2.19

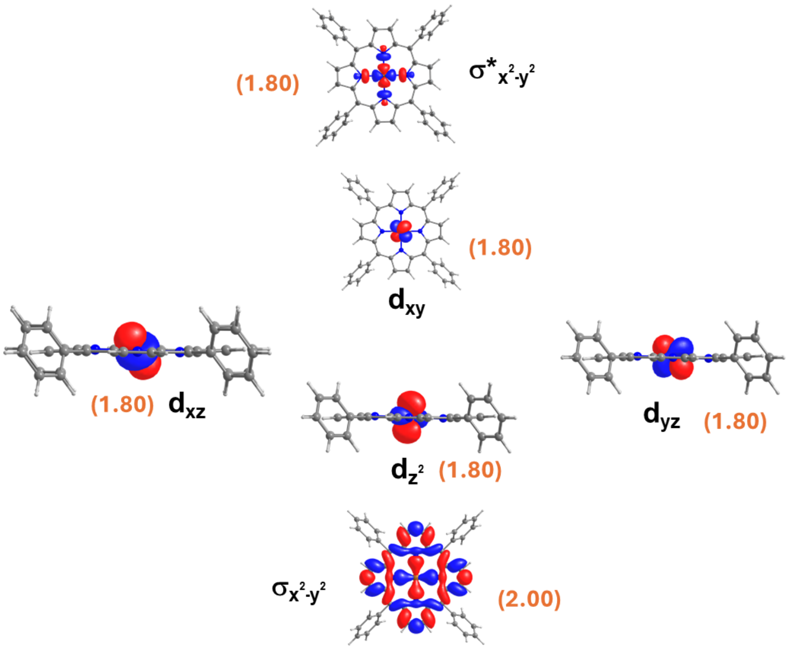

Figure S4. Pseudo-natural orbitals from SA(5)-CASSCF(11,6) calculations of the CuTPP molecule, along with the natural (average) orbital occupations.

Table S12: Vertical excited states obtained from the SA-CASSCF(17,12) calculations using 5 doublets for the CuTPP molecule.

Excited states	CASSCF Wave function (major weightage)<br>$\pi(p)\pi(p)\ \pi(p)\ \pi(p)\ d_{z^2} d_{xz}\ d_{yz}\ d_{xy}\ d_{x^2-y^2}\ \pi(p)^*\pi(p)^*\ \pi(p)^*$	SA-CASSCF energy (eV)
GS	2 2 2 2 2 2 2 2 2 1 0 0 0 (89%)	0.0
1st ES	2 2 2 2 2 2 2 2 1 2 0 0 0 (86%)	1.37
2nd ES	2 2 2 2 2 1 2 2 2 2 0 0 0 (86%)	1.56
3rd ES	2 2 2 2 2 2 1 2 2 2 0 0 0 (87%)	1.56
4th ES	2 2 2 2 1 2 2 2 2 2 0 0 0 (87%)	1.64

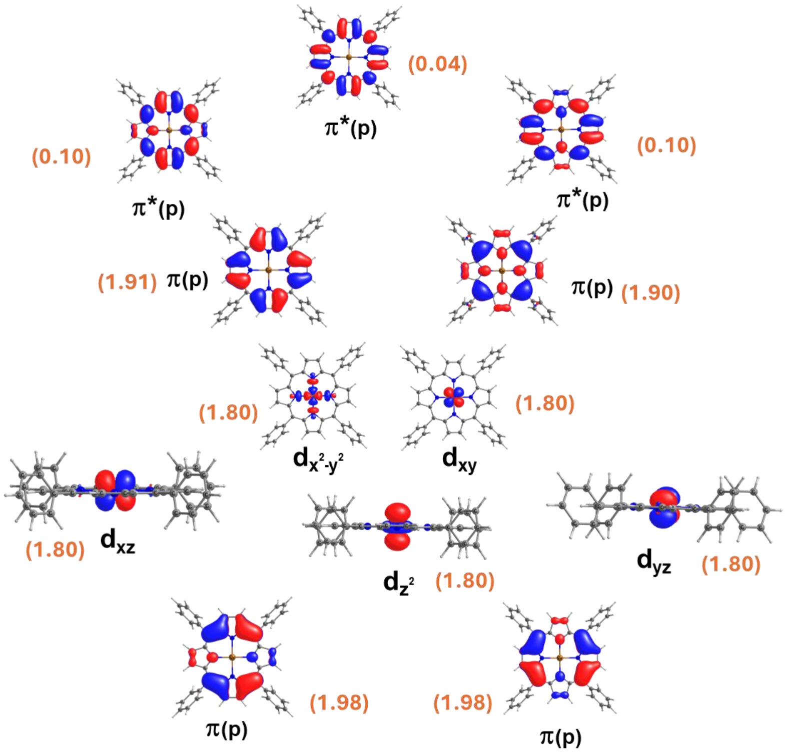

Figure S5. State-average (5 roots) canonical orbitals from SA-CASSCF(17,12) calculations of the CuTPP molecule. The electronic occupation corresponds to the pseudo-natural occupation number.

Table S13: Vertical excited states obtained from the SA-CASSCF(17,12) calculations using 12 doublets and 4 quartets for the CuTPP molecule. Only dominant configurations (determinants, *u*=up, *d*=down spin) are shown; the conjugate determinants are omitted for clarity.

Excited states	CASSCF Wave function (major weightage)<br>$\pi(p)\pi(p)\ d_{z^2} d_{xz} d_{yz} d_{xy}\ \pi(p)\pi(p)\ d_{x^2-y^2}\ \pi(p)^*\pi(p)^*\ \pi(p)^*$	SA-CASSCF (NEVPT2) energy (eV)	Bands (CASSCF Osc. Str.)	Exp. (eV)
GS	2 2 2 2 2 2 2 2 u 0 0 0 (88%)	0.0		
1st ES	2 2 2 2 2 u 2 2 2 0 0 0 (88%)	1.46 (2.03)		

2nd ES	2 2 2 2 u 2 2 2 2 0 0 0 (67%)<br>2 u 2 2 2 2 2 2 2 0 0 0 (21%)	1.72<br>(2.16)		
3rd ES	2 2 2 u 2 2 2 2 2 0 0 0 (67%)<br>u 2 2 2 2 2 2 2 2 0 0 0 (9%)	1.72<br>(2.17)		
4th ES	2 2 u 2 2 2 2 2 2 0 0 0 (88%)	1.81<br>(2.27)		
5th ES	2 2 2 2 2 2 2 u u d 0 0 (89%)	2.32<br>(2.13)	Q(10^{-5})	2.00
6th ES	2 2 2 2 2 2 2 u u 0 d 0 (89%)	2.35<br>(2.12)	Q(10^{-5})	
7th ES	2 2 2 2 2 2 u 2 u d 0 0 (88%)	2.69<br>(1.85)	Q(10^{-7})	
8th ES	2 2 2 2 2 2 u 2 u 0 d 0 (88%)	2.69<br>(1.84)	Q(10^{-7})	
9th ES	2 2 2 2 2 2 2 u u 0 d 0 (51%)<br>2 2 2 2 2 2 u 2 u d 0 0 (37%)	2.96<br>(2.16)	Q(0.03)	2.30
10th ES	2 2 2 2 2 2 2 u u d 0 0 (51%)<br>2 2 2 2 2 2 u 2 u 0 d 0 (36%)	2.98<br>(2.16)	Q(0.03)	
11th ES	2 2 2 2 2 u 2 u 2 d 0 0 (89%)	3.77<br>(4.15)		
Quartet states				
1st ES	2 2 2 2 2 2 2 u u u 0 0 (89%)	2.31<br>(2.12)		
2nd ES	2 2 2 2 2 2 2 u u 0 u 0 (89%)	2.34<br>(2.11)		
3rd ES	2 2 2 2 2 2 u 2 u u 0 0 (88%)	2.67<br>(1.82)		
4th ES	2 2 2 2 2 2 u 2 u 0 u 0 (88%)	2.67<br>(1.82)		

Coordinates:

Gas-phase optimized coordinates of H_2TPP (in Angstrom)

78
C 5.450087969 7.227838400 3.534892099
C 4.041120269 7.636485897 3.525420037
H 3.178339125 6.962046401 3.518040591
C 4.041091829 9.004264155 3.525238952
H 3.178281937 9.678659291 3.534532816
C 5.450040684 9.413126023 3.512717051
C 5.852361041 5.867564635 3.534585290
C 4.789854355 4.814804365 3.549671506
C 3.974226973 4.633688036 4.687984142

H	4.134078959	5.275788262	5.567355405
C	4.585645707	3.981367540	2.428360313
H	5.211370092	4.124989518	1.534350428
C	2.978542810	3.644652352	4.703591949
H	2.353398222	3.513697302	5.600291555
C	3.589944061	2.991972983	2.444587930
H	3.439415482	2.354568275	1.559651439
C	2.783485521	2.820618224	3.582232591
N	6.282804580	8.320534816	3.522965172
C	9.456561898	5.414120336	3.518751811
C	9.014506985	4.041235028	3.510928522
H	9.684031822	3.175824622	3.495398226
C	7.632572309	4.040263069	3.527942544
H	6.964255805	3.173886146	3.541161571
C	7.188660315	5.412514069	3.523409820
C	10.792407983	5.870393902	3.510480562
C	11.855817279	4.818464922	3.494898075
C	12.060129255	3.983523682	4.615066343
H	11.433604869	4.124941100	5.508883120
C	12.672103865	4.639614993	2.356761704
H	12.512195766	5.283015890	1.478340119
C	13.056700572	2.995105920	4.597883767
H	13.207345607	2.356413006	5.481862691
C	13.668728571	3.651484978	2.340129942
H	14.294323237	3.522567830	1.443440247
C	13.863967661	2.826078402	3.460382402
N	8.322058817	6.200550375	3.521886603
C	7.187437198	11.229880162	3.518753669
C	7.629493240	12.602765003	3.510923534
H	6.959970649	13.468177104	3.495385492
C	9.011428181	12.603735807	3.527945164
H	9.679745478	13.470112070	3.541163151
C	9.455338634	11.231484718	3.523414155
C	5.851590057	10.773607267	3.510484819
C	4.788181719	11.825536520	3.494901184
C	4.583897705	12.660509161	4.615051597
H	5.210442283	12.519114005	5.508857860
C	3.971867253	12.004357633	2.356779770
H	4.131753803	11.360933900	1.478370965
C	3.587329568	13.648929601	4.597865472
H	3.436708629	14.287647320	5.481830515
C	2.975244290	12.992489303	2.340145380
H	2.349628815	13.121383213	1.443466934
C	2.780034957	13.817927974	3.460379251
N	8.321939701	10.443449462	3.521891200
C	11.193910231	9.416161322	3.534890624
C	12.602877908	9.007514130	3.525412218
H	13.465658430	9.681954609	3.518031110

C	12.602906280	7.639736103	3.525222394
H	13.465715271	6.965339674	3.534505678
C	11.193957499	7.230874153	3.512710809
C	10.791637218	10.776434928	3.534588623
C	11.854144704	11.829194057	3.549679066
C	12.669765795	12.010308967	4.687996159
H	12.509907482	11.368208893	5.567366415
C	12.058360652	12.662629816	2.428368717
H	11.432640147	12.519009083	1.534355878
C	13.665452228	12.999342477	4.703608979
H	14.290592382	13.130296459	5.600311866
C	13.054064506	13.652022264	2.444601281
H	13.204598814	14.289426601	1.559665488
C	13.860517505	13.823375514	3.582250173
N	10.361193394	8.323465758	3.522962055
H	2.002400237	2.045116772	3.594819270
H	14.645754717	2.051297872	3.447107403
H	14.641604754	14.598875032	3.594840609
H	8.320899426	7.230108149	3.522356880
H	8.323096206	9.413891962	3.522359041
H	1.998249823	14.592710443	3.447101817

Gas-phase optimized coordinates for VOTPP

78
symmetry c1

V	8.322000000	8.322001000	2.978351000
C	5.452500000	7.218479000	3.505141000
C	4.067445000	7.642417000	3.540979000
H	3.204844000	6.968746000	3.562390000
C	4.070755000	9.016307000	3.549488000
H	3.212166000	9.694207000	3.591798000
C	5.457108000	9.434244000	3.497942000
C	5.863223000	5.871052000	3.500219000
C	4.807163000	4.812416000	3.510873000
C	4.015799000	4.594345000	4.659269000
H	4.189286000	5.211584000	5.553892000
C	4.588607000	4.010718000	2.369832000
H	5.199613000	4.182682000	1.470701000
C	3.027553000	3.597453000	4.665795000
H	2.421224000	3.435595000	5.570306000
C	3.599915000	3.014281000	2.377125000
H	3.437523000	2.400056000	1.478095000
C	2.816761000	2.804983000	3.524774000
N	6.288521000	8.324681000	3.477787000
O	8.321999000	8.322003000	1.406646000
C	9.425522000	5.452500000	3.505131000
C	9.001583000	4.067445000	3.540959000

H	9.675254000	3.204843000	3.562362000
C	7.627693000	4.070755000	3.549475000
H	6.949792000	3.212166000	3.591782000
C	7.209756000	5.457109000	3.497938000
C	10.772949000	5.863223000	3.500214000
C	11.831585000	4.807163000	3.510865000
C	12.049644000	4.015785000	4.659253000
H	11.432397000	4.189262000	5.553873000
C	12.633293000	4.588618000	2.369829000
H	12.461339000	5.199634000	1.470703000
C	13.046535000	3.027537000	4.665778000
H	13.208383000	2.421197000	5.570284000
C	13.629729000	3.599925000	2.377120000
H	14.243962000	3.437542000	1.478094000
C	13.839015000	2.816757000	3.524762000
N	8.319320000	6.288522000	3.477785000
C	7.218478000	11.191500000	3.505133000
C	7.642417000	12.576556000	3.540964000
H	6.968747000	13.439156000	3.562368000
C	9.016307000	12.573245000	3.549477000
H	9.694207000	13.431834000	3.591783000
C	9.434244000	11.186892000	3.497941000
C	5.871052000	10.780777000	3.500215000
C	4.812416000	11.836837000	3.510866000
C	4.594354000	12.628214000	4.659254000
H	5.211600000	12.454736000	5.553874000
C	4.010709000	12.055382000	2.369829000
H	4.182665000	11.444367000	1.470703000
C	3.597463000	13.616461000	4.665778000
H	3.435613000	14.222799000	5.570284000
C	3.014272000	13.044075000	2.377119000
H	2.400040000	13.206458000	1.478093000
C	2.804984000	13.827241000	3.524762000
N	8.324680000	10.355479000	3.477787000
C	11.191500000	9.425522000	3.505141000
C	12.576555000	9.001584000	3.540978000
H	13.439156000	9.675255000	3.562389000
C	12.573246000	7.627693000	3.549489000
H	13.431835000	6.949793000	3.591800000
C	11.186892000	7.209756000	3.497942000
C	10.780777000	10.772948000	3.500220000
C	11.836837000	11.831585000	3.510874000
C	12.628201000	12.049657000	4.659268000
H	12.454713000	11.432419000	5.553892000
C	12.055394000	12.633281000	2.369832000
H	11.444388000	12.461316000	1.470701000
C	13.616447000	13.046548000	4.665794000
H	14.222776000	13.208407000	5.570305000

C	13.044086000	13.629717000	2.377123000
H	13.206479000	14.243941000	1.478093000
C	13.827240000	13.839016000	3.524772000
N	10.355479000	8.319320000	3.477788000
H	14.620639000	2.041714000	3.530033000
H	14.602282000	14.620642000	3.530044000
H	2.041719000	2.023357000	3.530047000
H	2.023359000	14.602284000	3.530032000

Gas-phase optimized coordinates for CuTPP

77

Cu	0.000000000	0.000000000	0.000000000
N	2.033398061	-0.000000215	0.015937652
N	-0.000004560	-2.031505573	0.000190583
C	2.861452355	-1.105844685	0.074009851
C	2.452433064	-2.453101590	0.067318591
C	1.105575865	-2.861838288	0.030375479
C	4.246337242	-0.686118502	0.186349733
H	5.100999431	-1.364292590	0.275980675
C	0.685996939	-4.251008607	0.011506192
H	1.364089451	-5.110338451	0.015207579
C	3.510427159	-3.507903717	0.112335367
C	4.387798572	-3.691770776	-0.978491260
H	4.274744952	-3.053579111	-1.867973500
C	5.384449897	-4.679159119	-0.935482782
H	6.057662763	-4.813591454	-1.796156346
C	5.519180905	-5.497115449	0.198998098
C	4.651664297	-5.321842356	1.290381497
H	4.755197309	-5.955175222	2.184945599
C	3.654733784	-4.334807597	1.247742862
H	2.979833044	-4.188342138	2.104646412
N	-2.033399892	-0.000000196	-0.015564249
C	-2.861461958	-1.105844005	-0.073535126
C	-2.452444700	-2.453101387	-0.066831303
C	-1.105586306	-2.861838782	-0.029919756
C	-4.246353634	-0.686118154	-0.185789285
H	-5.101023879	-1.364292600	-0.275340275
C	-0.686007602	-4.251009509	-0.011011193
H	-1.364100422	-5.110339312	-0.014658785
C	-3.510443487	-3.507902732	-0.111753227
C	-4.387763383	-3.691721136	0.979122948
H	-4.274665532	-3.053492255	1.868572811
C	-5.384419782	-4.679108204	0.936203609
H	-6.057591877	-4.813502361	1.796915023
C	-5.519207826	-5.497111440	-0.198236676
C	-4.651743399	-5.321886247	-1.289669260

H	-4.755321624	-5.955255754	-2.184202161
C	-3.654807792	-4.334852822	-1.247119694
H	-2.979948225	-4.188424700	-2.104062011
N	-0.000004536	2.031505565	0.000190590
C	-2.861462431	1.105843486	-0.073534678
C	-2.452445716	2.453101637	-0.066830449
C	-1.105586788	2.861839708	-0.029919277
C	-4.246353960	0.686117155	-0.185789017
H	-5.101024719	1.364291545	-0.275339635
C	-0.686007775	4.251010617	-0.011010888
H	-1.364100915	5.110340980	-0.014658279
C	-3.510445223	3.507902696	-0.111751469
C	-4.387765467	3.691719596	0.979125154
H	-4.274667164	3.053489811	1.868574731
C	-5.384422473	4.679106236	0.936206652
H	-6.057594631	4.813499335	1.796918262
C	-5.519211093	5.497110309	-0.198232985
C	-4.651746841	5.321886636	-1.289666003
H	-4.755325838	5.955256839	-2.184198456
C	-3.654810481	4.334853736	-1.247117620
H	-2.979950960	4.188426616	-2.104060702
C	2.861452829	1.105844120	0.074009370
C	2.452434130	2.453101794	0.067317675
C	1.105576418	2.861839193	0.030374932
C	4.246337565	0.686117476	0.186349427
H	5.101000273	1.364291509	0.275979995
C	0.685997241	4.251009716	0.011505658
H	1.364090101	5.110340119	0.015206689
C	3.510428959	3.507903621	0.112333500
C	4.387800022	3.691769862	-0.978494037
H	4.274745456	3.053577785	-1.867976285
C	5.384451865	4.679157867	-0.935486502
H	6.057664204	4.813589692	-1.796160646
C	5.519184114	5.497114413	0.198994103
C	4.651668451	5.321842094	1.290378431
H	4.755202784	5.955175126	2.184942399
C	3.654737256	4.334807781	1.247741079
H	2.979837188	4.188342724	2.104645791
H	-6.300888115	-6.271393987	-0.232007116
H	6.300857051	-6.271399123	0.232838316
H	-6.300891952	6.271392342	-0.232002685
H	6.300860771	6.271397641	0.232833503
